# Recent insights on principles of synaptic protein degradation

**DOI:** 10.12688/f1000research.10599.1

**Published:** 2017-05-15

**Authors:** Laurie D. Cohen, Noam E. Ziv

**Affiliations:** 1 Technion Faculty of Medicine, Rappaport Institute and Network Biology Research Laboratories, Technion City, Haifa, 32000, Israel

**Keywords:** synaptic protein degradation, autophagy, canonical degradation pathways, ubiquitin-proteasome system, TS:YFP, PKMζ, PKCλ

## Abstract

Maintaining synaptic integrity and function depends on the continuous removal and degradation of aged or damaged proteins. Synaptic protein degradation has received considerable attention in the context of synaptic plasticity and growing interest in relation to neurodegenerative and other disorders. Conversely, less attention has been given to constitutive, ongoing synaptic protein degradation and the roles canonical degradation pathways play in these processes. Here we briefly review recent progress on this topic and new experimental approaches which have expedited such progress and highlight several emerging principles. These include the realization that synaptic proteins typically have unusually long lifetimes, as might be expected from the remote locations of most synaptic sites; the possibility that degradation pathways can change with time from synthesis, cellular context, and physiological input; and that degradation pathways, other than ubiquitin-proteasomal-mediated degradation, might play key roles in constitutive protein degradation at synaptic sites. Finally, we point to the importance of careful experimental design and sufficiently sensitive techniques for studying synaptic protein degradation, which bring into account their slow turnover rates and complex life cycles.

## Introduction

Communication between neurons in the brain is based primarily on chemical synapses, minute and complex cellular devices that transfer signals from one cell to another through timed neurotransmitter secretion and reception. Synapses are composed of hundreds of proteins which have finite lifetimes; consequently, maintaining synaptic integrity and function depends on the continuous removal and degradation of aged or damaged proteins and their replacement with freshly synthesized copies.

How are synaptic proteins degraded? In many respects, this is an ill-posed question. First, synaptic proteins belong to diverse structural and functional classes. Second, they often have complex life cycles, during which they move through cellular compartments (e.g. the endoplasmic reticulum, the Golgi apparatus, the plasma membrane, and vesicular organelles), amid cellular regions (e.g. cell bodies and remote axonal/dendritic sites), between synaptic and extrasynaptic sites, and between multi-molecular complexes and free pools. The manner by which synaptic proteins are degraded is thus likely to vary not only between different molecules but also between different contexts and locations (e.g. ^1^; see also ^2^). Furthermore, synaptic protein degradation can be affected by physiological signals (e.g. activity levels) or altered by pathological processes. In the former context, the effects of physiological signals on synaptic protein degradation have been mainly studied in relation to synaptic plasticity (reviewed in ^2– 10^). In the latter context, neuronal protein degradation has been studied mainly in relation to neurodegenerative conditions such as Alzheimer’s (e.g. ^11, 12^), Parkinson’s (e.g. ^13^), and Huntington’s disease ^14^ as well as other neurological diseases ^10^ (see also ^5, 15– 20^ for additional reviews). Somewhat surprisingly, much less attention has been given to constitutive, ongoing synaptic protein degradation. Here we wish to focus mainly on this topic and to highlight emerging principles based on recent progress and new experimental approaches.

## Synaptic protein lifetimes

A first step toward understanding the manners by which synaptic proteins are degraded is to have a good grasp of their lifetimes: protein synthesis ^21^, processing, and, in particular, trafficking to (and from) remote synapses (e.g. ^22– 24^) takes time which can amount to many hours or days. Lifetimes thus provide important clues to the life cycle of synaptic proteins ^25^. For example, if the lifetime of a presynaptic protein is in the order of a few hours, this would imply local synthesis and degradation, simply because of the incompatibility of such short lifetimes with processing and trafficking rates (reviewed in ^26^). Similarly, comparisons of the lifetimes of related proteins (e.g. synaptic vesicle proteins) can hint at whether these are degraded in bulk (for instance, through degradation of entire synaptic vesicles) or, alternatively, processed separately ^27^.

Unfortunately, estimates of synaptic protein lifetimes, even for the same proteins, vary substantially. Older studies based on radiolabeling methods indicated that presynaptic proteins can be degraded at remarkably slow rates, exhibiting half-lives of many days and even weeks, e.g. ^28, 29^ (see also ^26^), as might be expected from known trafficking rates and the extraordinary lengths of some axons. Conversely, other studies indicated that the lifetimes of some synaptic proteins, mainly postsynaptic (e.g. ^30^) but also presynaptic ^31^, might be much shorter, with half-lives in the order of several hours, with yet other studies reporting intermediate values (reviewed in ^2^).

Recent studies, based on metabolic labeling methods combined with mass spectroscopy (MS) and on newly developed fluorescent reporters, are starting to provide realistic estimates and hints as to the source of some discrepancies. In one such study ^32^, rat cortical neurons grown in culture for 2 weeks were exposed to amino acids containing stable heavy isotopes of carbon and nitrogen; by resolving the incorporation and loss rates of labeled and unlabeled amino acids, respectively, degradation rates for many hundreds of neuronal and synaptic proteins were estimated. This study suggested that half-lives of synaptic proteins in these preparations are in the order of 2 to 5 days, with some synaptic proteins exhibiting even longer half-lives (findings verified and extended in a subsequent study ^33^). Somewhat unexpectedly, metabolic turnover rates of presynaptic and postsynaptic proteins were not significantly different, nor did these differ substantially from proteins for which mRNAs are consistently found in dendrites. Using the mean synaptic protein half-life value obtained in the former study (4.14 days), the authors estimated that, on average, ~0.7% of the synaptic protein content in neurons is replaced every hour (for details, see ^32^).

Interestingly, an earlier analysis of proteome dynamics by means of organism-wide isotopic labeling and MS ^27^ suggests that, in the adult brain, degradation rates are even slower; in fact, comparing half-life estimates for 467 proteins and 90 synaptic proteins resolved in both data sets suggests that half-lives in the adult mouse brain are, on average, ~2.7-fold longer, ranging from ~1.5 days to ~48 days for these 90 synaptic proteins (average ≈12 days, with presynaptic proteins exhibiting a trend for longer half-lives). Comparable lifetimes for some synaptic proteins were also reported in a subsequent organism-wide isotopic labeling study ^34^.

Half-life estimates provided in these studies were, in some cases, very different from estimates based on traditional pulse-chase labeling with radioactive substrates (such as ^35^S cysteine or methionine). It is important to note, however, that labeling periods in traditional pulse-chase experiments tend to be relatively short (20 to 60 minutes, e.g. ^35– 38^; see ^2^ for a more comprehensive listing), running the risk of biasing estimates towards protein pools with fast turnover rates. In this regard, a recent metabolic labeling/MS study shows compellingly that for a substantial number of proteins, a disproportionally large amount of newly synthesized protein is degraded within hours of its synthesis ^39^. Here stable isotope labeling was combined with biorthogonal amino acid labeling ^40^ to identify newly synthesized proteins (see also ^41^). It was found that >10% (possibly more) of proteins expressed by two different cell lines exhibited non-exponential degradation kinetics, i.e. rapid degradation during the first hours of their lives, followed by much slower degradation thereafter. This recent study joins prior studies (e.g. ^42, 43^) pointing to rapid degradation of substantial numbers of newly synthesized protein copies owing to a variety of reasons: failure to fold properly (e.g. ^44– 46^, reviewed in ^47^), superstoichiometric synthesis, and subsequent failure to integrate into appropriate complexes (the “stabilized binding complex” model ^48^—see also ^43^—or as regulatory steps in determining cellular contents of key proteins ^49, 50^). If these findings apply to synaptic proteins (which tend to assemble into multimolecular complexes), this would reinforce the notion that questions on synaptic protein degradation must be refined to consider their status in terms of life cycle stage and cellular location.

Synaptic protein degradation rates are also starting to provide clues regarding a lingering question: do synaptic vesicles maintain their complement of molecular constituents throughout multiple exocytosis and endocytosis cycles, or are they continuously reformed and their contents mixed? If the former, lifetimes of synaptic vesicle proteins might be expected to be rather similar. This does not seem to be the case, however, as lifetimes of synaptic vesicle proteins vary widely ^32, 51^. Rather, the heterogeneity in degradation rates would seem to suggest that synaptic vesicle proteins are probably intermixed and sorted for degradation in ways that are at least partly independent of each other, as described later on. The possibility remains, however, that such differences could stem, at least in part, from the existence of distinct vesicle pools with distinct protein compositions.

Although metabolic labeling techniques coupled to MS can provide rich information on protein degradation rates and pathways, these techniques are strongly biased toward relatively abundant proteins; consequently, the applicability of their conclusions to less abundant proteins remains unclear (but see ^48^). Moreover, their resolving power in terms of life cycle stage and cellular location is limited, even when fractionation techniques are used during preparative steps. A very different approach, based on cellular imaging and fluorescent reporters, was recently shown to allow high spatial and temporal resolution measurements of specific synaptic proteins ^52, 53^. The fluorescent reporters developed for this purpose (time-specific tag for the age measurement of proteins [TimeSTAMP] ^54^) are cleverly designed constructs containing a viral protease flanked by cognate protease sites and two “halves” of split yellow fluorescent protein (YFP). By default, the protease excises itself, resulting in non-fluorescent protein fragments which are rapidly degraded. Applying a cell-permeant protease inhibitor, however, prevents excision and allows stable complementation of the flanking regions into functional YFP. Fusing these constructs, known as TimeSTAMP:YFP (TS:YFP) to target proteins allows for the visualization of newly synthesized copies of these proteins or, by removing the inhibitor, the visualization of their degradation. This technique has been used to measure degradation rates of neuroligin-3 ^52^ as well as protein kinase Mζ (PKMζ) and PKCλ in somata and dendritic spines ^53^. It should be noted, however, that this method reports degradation rates of fusion proteins expressed via non-native promoters, which might differ from those of native proteins. Thus, for example, the half-life of neuroligin-3:TS:YFP was estimated as 24 hours ^52^ as compared to stable isotope labeling-based estimates for native neuroligin-3 of 66 ^32^ and 63 ^33^ hours.

A new method that overcomes some of these limitations was recently described ^55^. This method, based on a combination of biorthogonal amino acid labeling/puromycylation, specific antibodies, and a proximity ligation assay, provides a sensitive means for visualizing and measuring the synthesis and degradation rates of endogenous synaptic proteins *in situ*. So far, the method has been used to measure the degradation rates of the neurotrophin receptor TrkB and the presynaptic active zone protein Bassoon, revealing degradation rates that are comparable with previously published rates ^32^. Unfortunately, the method requires fixation and is thus unsuitable for live imaging. Furthermore, the small fields of view typical of imaging approaches pose difficulties in separating *bona fide* degradation from trafficking of labeled proteins out of imaged regions. Yet, given that this difficulty can be resolved by whole neuron imaging (e.g. ^56^), this new method shows great promise.

In summary, while there is still much to learn on synaptic protein lifetimes, it would seem safe to say that the lifetimes of many synaptic proteins are relatively long (days) and compatible with constraints imposed by distances of synaptic sites from central protein synthesis (and degradation) systems.

## Canonical degradation pathways and synaptic protein degradation

### The ubiquitin-proteasome system

Where canonical protein degradation pathways are concerned, synaptic protein degradation via the ubiquitin-proteasome system (UPS) has undoubtedly received the most attention. Indeed, a significant number of synaptic proteins have been shown to undergo ubiquitination and/or degradation in a ubiquitination-dependent manner (e.g. ^31, 37, 49, 57– 67^). Moreover, pharmacological suppression of proteasomal activity has been shown to affect global and/or synaptic levels of synaptic proteins ^30, 49, 50, 57, 68, 69^ (reviewed in ^2, 5, 70– 72^). Yet the interpretation of such findings is not always straightforward. First, it often remains unknown at what point during a synaptic protein’s life cycle ubiquitination-dependent degradation occurs, a matter that cannot be ignored given the central role of UPS-mediated degradation in quality-control processes (see below). Second, ubiquitination does not necessarily imply degradation, as ubiquitination can act as a signal for other downstream events or as an effector of protein function without affecting synaptic protein levels ^73– 75^ (see also ^76^). Third, under physiological conditions, ubiquitination is continuously countered by deubiquitinating enzymes; thus, ubiquitination cannot be taken as unequivocal evidence for imminent proteasomal-based degradation ^77, 78^. Fourth, ubiquitination can also mark proteins for lysosomal degradation or autophagy (see below). Finally, many of these findings are based, at least in part, on pharmacological agents that inhibit proteasomal activity; proteasome inhibition, however, can lead to unexpected results, including suppressed synthesis of most proteins and enhanced synthesis of others (e.g. ^1, 33, 79– 85^). Unfortunately, methods typically used for studying the effects of proteasomal inhibition (e.g. Western blots and quantitative immunohistochemistry) are incapable of separating effects on protein degradation from effects on protein synthesis, as they report *total* protein quantities. In part, this problem can be bypassed by pharmacologically inhibiting protein synthesis; this manipulation, however, severely limits experiment duration and has been shown to affect protein degradation pathways (e.g. ^86^). Pulse-chase experiments based on radioactive amino acids are better in this respect but, as mentioned above, are not well suited for studying the degradation of long-lived proteins because of the conflicting requirements for long labeling periods and low concentrations of unlabeled amino acids, and the compromised availability of essential amino acids this entails.

**Figure 1.**
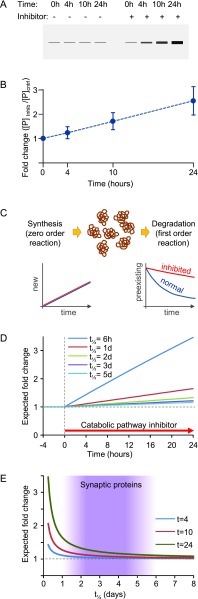
Measuring the effects of catabolic inhibitors on synaptic protein degradation is challenged by the slow turnover rates of most synaptic proteins. **A**) In a typical experiment, total protein content is measured (by Western blots, for example) following exposures to a pharmacological inhibitor (e.g. MG-132) of a degradation pathway (e.g. the ubiquitin-proteasome system [UPS]) for increasing durations. **B**) The fold-change is then plotted as a function of time, giving rise to plots such as that shown here. **C**) In the most simple view of synaptic protein metabolism, 1) protein synthesis occurs at a constant rate, 2) protein degradation occurs as a first-order reaction with a rate coefficient of $\approx \frac{1}{1.44\cdot t_{\text{1/2}}}$ where t _½_ is the half-life of the protein of interest, 3) at steady state, the rate of protein synthesis is equal to the rate of protein degradation, resulting in constant protein concentrations, and 4) a pharmacological catabolic pathway inhibitor strongly reduces the degradation rate coefficient but does not affect the rate of protein synthesis. **D**) Under these assumptions, expected fold-changes in protein levels are shown for five proteins with increasingly longer half-lives. Note the minuscule changes for proteins with half-lives of 3 days or longer. The inhibitor is assumed to reduce the degradation rate coefficient by a factor of 20 (greater reductions do not significantly change these results). **E**) The expected fold-change in total protein content as a function of protein half-life is shown for three inhibition durations (4, 10, or 24 hours). The shaded region represents the half-lives of the majority of synaptic proteins for which half-life estimates were obtained in a prior study ^32^. Note that even after inhibiting the catabolic pathway of interest for 24 hours, and even when assuming that protein synthesis rates are not reduced, expected changes in total synaptic protein amounts are very modest, raising a requirement for highly sensitive and accurate quantification methods and demonstrating the importance of prior knowledge regarding turnover rates for correctly interpreting results in such experiments.

In a recent study ^33^, metabolic labeling with stable isotopes was used to specifically measure the degree to which proteasomal inhibition slowed the degradation of hundreds of proteins expressed by cultured rat cortical neurons using an approach that overcame most of the aforementioned limitations. Somewhat surprisingly, highly effective proteasomal inhibitors did not slow the degradation of most identified proteins (1,530, including 176 synaptic proteins in total). In other words, apart from relatively small sets of neuronal and synaptic proteins, degradation of most identified proteins continued at normal rates, even when proteasome-mediated degradation was effectively inhibited. This analysis, however, also illustrated the inherent limitations of probing synaptic protein degradation through catabolic inhibition ( Figure 1). Given the slow degradation rates of many synaptic proteins, short periods of catabolic inhibition might barely affect protein abundance. As an example, consider a protein with a half-life of 7 days (such as the postsynaptic density [PSD] protein ProSAP1/Shank2 ^32^). Here, inhibiting its catabolism for 4, 10, or 24 hours would be expected to increase its abundance by merely ~1.6%, 4%, and 9%, respectively. Thus, without prior knowledge of the half-lives of the proteins of interest, as well as a good appreciation of measurement sensitivity, results of such experiments remain inconclusive. In the aforementioned study ^33^, known half-lives for identified proteins were used to calculate the expected impact of catabolic inhibition, and these values were compared with experimental measurements and constrained by calibrations of measurement sensitivity. This analysis allowed the authors to conclude that proteasomal inhibitors did not slow the degradation of many, if not most synaptic proteins, although findings remained inconclusive for some synaptic proteins whose particularly long half-lives (>5 days) precluded sufficiently accurate measurements.

Although these findings might seem surprising at first, they are in good agreement with prior large-scale studies carried out in human colon cancer ^77^, HeLa ^87^, human Jurkat ^78^, and osteosarcoma cells ^1^. In all of these studies, practically no changes in the abundance of thousands of proteins were observed following 5–8 hours of proteasomal inhibition, in spite of dramatic changes in protein ubiquitination ^77, 87^.

A possible complication in the interpretation of these studies relates to the consistent observation that prolonged proteasomal inhibition induces proteotoxic stress, driven by the buildup of unfolded/misfolded proteins ^88, 89^. This stress, in turn, triggers cellular responses, such as the unfolded protein response (UPR), which leads to a generalized shutdown of protein synthesis (reviewed in ^47^). Interestingly, the synthesis of synaptic proteins appears to be particularly sensitive to this response ^33^ (see also ^90^ for a recent review on relationships between the UPR and neurodegeneration), further confounding interpretations based on total protein abundance measurement in proteasomal inhibition experiments.

What is now clear beyond doubt is the crucial role the UPS plays in rapidly degrading newly synthesized, misfolded ^47^, excess, or superstoichiometric proteins ^39^. It is thus possible that UPS-mediated degradation of some synaptic proteins occurs primarily during the earliest phases of their life cycle, namely immediately after synthesis and initial processing (see above). In fact, for both γ-Aminobutyric acid (GABA) _A_^49^ and GABA _B_ receptors ^50^, this was proposed to serve as an important regulatory step. Conversely, UPS-mediated degradation might play a secondary role once the proteins are localized within synapses, at least under baseline conditions. In agreement with this possibility, it was suggested that some recently synthesized, particularly large, proteins are degraded rapidly after their synthesis in a UPS-dependent manner and at rates unexpected from their known lifetimes ^33^. Along these lines, a recent electron cryotomography study suggests that in the absence of proteotoxic stress, only 20% of 26S proteasomes in cultured hippocampal neurons are actively engaged in substrate processing, which might be taken as evidence for their importance in handling excess or misfolded proteins ^91^.

This proposition does not negate the importance of locally acting, activity-regulated, UPS-mediated synaptic protein degradation ^2– 8^. Thus, for example, a series of studies has demonstrated and characterized the redistribution of proteasomes to dendritic spines following synaptic stimulation or activation of postsynaptic N-methyl-D-aspartate (NMDA)-type glutamate receptors ^92– 96^. Along these lines, NMDA receptor activation was shown to drive PSD remodeling through UPS-mediated degradation of PSD-95 (a major postsynaptic scaffolding protein ^68^). Similarly, an activity-inducible kinase (serum-inducible kinase) was shown to mark UPS-mediated degradation of another PSD protein (spine-associated Rap GTPase-activating protein), leading to subsequent synapse loss ^97^. Interestingly, the degradation of this kinase was also found to be UPS mediated. Changes in activity levels were also shown to drive UPS-mediated degradation of the postsynaptic scaffolding protein GKAP/SAPAP ^66^, although, in this case, the actual degradation of synaptic GKAP seems to occur only after transporting it away from synapses to centralized locations. Interestingly, local proteasomal activity also seems to play important roles at very early stages of synapse formation, namely during the extension of nascent dendritic spine precursors ^98^. On the presynaptic side, UPS-mediated degradation was implicated in activity-dependent changes in global and synaptic levels of Rim and Munc-13 ^99^ and in global levels of Bassoon and liprin-α ^69^. Intriguingly, the large presynaptic active zone proteins Bassoon and Piccolo have been shown to locally suppress the ubiquitination and degradation of multiple presynaptic proteins, possibly by directly regulating the activity of the E3 ligase Siah1 ^100^ (see also ^75^).

While local, activity-regulated UPS-mediated degradation of certain synaptic proteins seems to be functionally important, this form of degradation does not generalize to many other synaptic proteins ^2, 5, 30, 66, 69, 99^. Moreover, the degree to which such local processes affect synaptic protein contents under basal conditions appears to be quite modest. For example, in spontaneously active primary cultures of mouse and rat cortical neurons, pharmacologically suppressing proteasomal function for 10–24 hour did not increase synaptic levels of PSD-95 and PSD-93 (24 hours ^66^), Munc13-1 (10 hours ^101^), PSD-95, Shank3/ProSAP2, SV2A, Synapsin I, or Bassoon (10–24 hours ^33^) and only slightly increased synaptic Rim levels ^33^ (incidentally, the half-life of Rim1 was estimated here to be ~3 days, which is more compatible with the spatiotemporal constraints discussed above as compared to a prior estimate of 0.7 hours ^31^). Finally, as mentioned above, suppression of proteasomal activity did not significantly slow constitutive degradation rates of most synaptic proteins ^33^. It is thus possible that local, activity-regulated UPS-mediated degradation mainly serves to shape synaptic properties in spatially and temporally constrained manners ^25^, with the constitutive degradation of most synaptically residing proteins relegated to other pathways.

### Autophagy and lysosomal degradation

An alternative catabolic route, which is receiving increasing attention in the context of synaptic protein degradation, is autophagy. A major form of autophagy is macroautophagy, a primary mechanism used by cells to degrade cytosolic complexes and membranous organelles such as mitochondria. Here, double-membraned structures engulf portions of cytoplasm to form autophagosomes, which ultimately fuse with lysosomes, where their contents are degraded. Mutant mice deficient for autophagy-specific genes in the nervous system (e.g. ^102– 105^) suffer from progressive neurological deficits, accumulation of abnormal cytoplasmic inclusions in neurons, and ultimately neuron loss and premature death. Along these lines, macroautophagy has been suggested to play important roles in removing cytosolic components and membranous organelles from remote axonal regions and targeting them for somatic degradation ^106– 108^. Similarly, roles for autophagy in presynaptic proteostasis, axonal membrane homeostasis (e.g. ^104, 109– 113^), and even degradation of entire synaptic vesicles (“vesiculophagy”) have been suggested ^114^. Finally, the active zone protein Bassoon, previously shown to locally suppress UPS-mediated synaptic protein degradation ^100^ (see above), has recently been shown to also control presynaptic autophagy through interactions with Atg5, an E3-like ligase essential for autophagy ^115^.

On the postsynaptic side, macroautophagy has been implicated in (activity-induced) degradation of a number of synaptic proteins (reviewed in ^116^) such as α-amino-3-hydroxy-5-methyl-4-isoxazolepropionic acid (AMPA)-receptor subunits ^117^, whereas genetic suppression of macroautophagy was shown to lead to an excess of excitatory synapses ^118^. Interestingly, macroautophagy has been studied mainly in the context of nutrient starvation and other forms of stress, but neither starvation nor pharmacological inhibition of mammalian target of rapamycin seem to induce overt macroautophagy in neurons ^107^ (but see ^117^). This would be congruent with a constitutive role for macroautophagy in synaptic proteostasis ^107^ (see also ^119^; reviewed in ^15^), although the flux and identities of synaptic proteins or protein complexes degraded in this fashion remain largely unknown.

A related degradation pathway is endosomal microautophagy, a process through which late endosomes directly engulf cytoplasmic material. A recent study suggests that this process may play a key role in the degradation of numerous synaptic proteins, at least in *Drosophila* ^120^. In this remarkable study, the chaperone Hsc70-4, initially identified by its ability to deform membranes, was shown to play a crucial role in microautophagy and degradation of Comt/NSF, Unc-13, and EndophilinA and the formation of multivesicular bodies (MVBs) containing multiple synaptic vesicles. Furthermore, a bioinformatic survey of about 170 rat synaptic proteins suggested that 53% of these contain at least one microautophagy motif. The involvement of Hsc70-4 in this process is interesting not only because of its function as a synaptic chaperone but also because it is a central player in a third type of autophagy known as chaperone-mediated autophagy (CMA), in which complexes of Hsc70 and target proteins are selectively translocated across the lysosomal membrane and subsequently degraded. Although CMA was deemed unlikely in *Drosophila* ^120^, the possibility that CMA as well as microautophagy play important roles in mammalian synaptic protein degradation remains intriguing ^16^.

Another interesting finding in the aforementioned study concerns the fate of synaptic transmembrane proteins. No evidence was found for microautophagy-mediated degradation of three such proteins (Synaptotagmin1, Syntaxin1A, and the vesicular glutamate transporter Vglut). This, and the observation that synaptic transmembrane proteins harbor fewer microautophagy motifs, led the authors to suggest that synaptic transmembrane protein degradation is mediated by “classical” lysosomal pathways, in agreement with prior studies regarding synaptic vesicle-associated protein degradation in flies ^121, 122^. Here, evidence was provided that endosomes served as sorting stations for synaptic vesicle proteins endocytosed as part of the synaptic vesicle cycle, sending dysfunctional proteins to lysosomes for degradation. Furthermore, the removal of such presumably dysfunctional proteins was associated with improved presynaptic functionality, a conclusion later supported by a study on the consequences of impaired retrograde transport of presynaptic endosomal cargos ^123^. Targeting to lysosomes seems to involve ubiquitination and the formation of MVBs whose formation depends on the endosomal sorting complex required for transport machinery. The universality of this presynaptic protein sorting and degradation process is supported by a recent study carried out in rat hippocampal neurons ^51^. Intriguingly, this study suggests that different synaptic vesicle proteins are degraded at different rates (SV2, Synaptotagmin1 » VAMP2 » Synaptophysin, Vglut1) and in manners differentially affected by manipulations of network activity levels, arguing against the turnover of synaptic vesicles as discrete units (as discussed above) and further highlighting the importance of sorting processes in the specific regulation of particular synaptic protein degradation.

On the postsynaptic side, considerable evidence suggests that AMPA receptors (transmembrane synaptic proteins) are continuously cycled between the plasma membrane and endosomal compartments; interestingly, all four AMPA-receptor subunit types (GluA1–4) can undergo activity-dependent ubiquitination, which is thought to determine whether internalized AMPA receptors are targeted for lysosomal degradation or recycled back to the membrane (reviewed in ^124^; see also ^125^). Multiple studies have demonstrated a crucial role for the E3 ligase Nedd4 in this form of regulatory ubiquitination ^63, 126– 129^. Proteasomal inhibitors were shown to strongly slow Nedd4 degradation rates ^33^, perhaps explaining the prominent effects of proteasomal inhibitors on AMPA receptor surface expression ^130^.

In common with AMPA receptors, ubiquitination of GABA _A_ receptors (more specifically, their γ subunits) seems to determine whether internalized GABA _A_ receptors are targeted for lysosomal degradation or recycled back to the membrane ^131^. GABA _B_ receptor degradation seems to follow a similar route ^132^. Collectively, these and additional findings point to the importance of endosomal sorting and lysosomal degradation in the catabolism of synaptic transmembrane proteins, in particular synaptic vesicle proteins, postsynaptic receptors, and possibly others (e.g. ^133, 134^; reviewed in ^70^).

## Summary

Recent studies, along with the advent of new technologies, have provided a plethora of data on synaptic protein degradation. Although much of it is undoubtedly confusing, several principles seem to be emerging. First, synaptic proteins have unusually long lifetimes, as might be expected from the challenging spatiotemporal constraints imposed by their remote locations. Second, the typically complex synaptic protein life cycle might imply multiple degradation routes that change with the protein's "age" (time from synthesis), cellular context, and physiological input. Third, despite the importance attributed to UPS-mediated degradation, alternative degradation pathways, perhaps ones that are specifically tailored to degrading large multimolecular complexes, might play key roles in ongoing synaptic protein degradation at synaptic sites. Finally, reliable experiments in this field call for experimental design and sensitive techniques that are well matched to the inherently slow rates of synaptic protein degradation and their complex life cycles.

One last emerging “principle” is that synaptic protein degradation might turn out to be immensely complex: first, because canonical degradation pathways do not act in isolation and often involve significant crosstalk (reviewed in ^135, 136^); second, because suppressing one degradation pathway (e.g. the proteasome) can, in some cases, elevate activity in other pathways (e.g. autophagy; ^135^, but see ^137^); and, finally, because of the existence of additional routes for synaptic protein degradation which involve calpains ^138^ and extracellular acting proteases (e.g. tissue plasminogen activator, β-site amyloid precursor protein-cleaving enzyme 1, and matrix metalloproteinases; reviewed in ^139, 140^ among others ^141^).

Given the life span of neurons and synapses, and the spatiotemporal challenges synapses face, we feel that even before considering issues of synaptic plasticity and neurodegenerative disorders, principles of constitutive synaptic protein degradation represent a fascinating yet poorly understood biological problem. Hopefully the plethora of new approaches and tools will help to uncover such principles.

## Abbreviations

AMPA, α-amino-3-hydroxy-5-methyl-4-isoxazolepropionic acid; CMA, chaperone-mediated autophagy; GABA, γ-Aminobutyric acid; MS, mass spectroscopy; MVB, multivesicular body; NMDA, N-methyl-D-aspartate; PK, protein kinase; PSD, postsynaptic density; TimeSTAMP, time-specific tag for the age measurement of proteins; TS:YFP, TimeSTAMP:yellow fluorescent protein; UPR, unfolded protein response; UPS, ubiquitin-proteasome system; YFP, yellow fluorescent protein.
